# Would induction of dopamine homeostasis via coupling genetic addiction risk score (GARS®) and pro-dopamine regulation benefit benzodiazepine use disorder (BUD)?

**DOI:** 10.15761/JSIN.1000196

**Published:** 2018-05-03

**Authors:** K Blum, M Gold, EJ Modestino, D Baron, B Boyett, D Siwicki, L Lott, A Podesta, AK Roy, M Hauser, BW Downs, RD Badgaiyan

**Affiliations:** 1 Western University Health Sciences, Graduate School of Biomedical Sciences, Pomona, CA, USA; 2 Division of Nutrigenomics, Geneus Health, LLC., San Antonio, TX, USA; 3 Division of Neuroscience & Addiction Research, Pathway Healthcare, LLc., Birmingham, AL, USA; 4 Division of Addiction Services, Dominion Diagnostics, LLC. North Kingstown, RI, USA; 5 Department of Psychiatry, Washington University School of Medicine, St. Louis, Mo, USA; 6 Department of Psychology, Curry College, Milton, MA, USA; 7 Department of psychiatry, Tulane University School of Medicine, New Orleans, LA, USA; 8 Division of Nutrigenomic Research, Victory Nutrition International, Lederach, PA, USA; 9 Department of Psychiatry, Veterans Administration Hospital at San Antonio, San Antonio, TX, USA

**Keywords:** benzodiazepines, benzodiazepine use disorder, dopaminergic, reward deficiency syndrome, gars, kb220, dopamine homeostasis

## Abstract

Prescriptions for Benzodiazepines (BZDs) have risen continually. According to national statistics, the combination of BZDs with opioids has increased since 1999. BZDs (sometimes called “benzos”) work to calm or sedate a person by raising the level of the inhibitory neurotransmitter GABA in the brain. In terms of neurochemistry, BZDs act at the GABAA receptors to inhibit excitatory neurons, reducing VTA glutaminergic drive to reduce dopamine release at the Nucleus accumbens. Benzodiazepine Use Disorder (BUD) is very difficult to treat, partly because BZDs are used to reduce anxiety which paradoxically induces hypodopaminergia. Considering this, we are proposing a paradigm shift. Instead of simply targeting chloride channel direct GABAA receptors for replacement or substitution therapy, we propose the induction of dopamine homeostasis. Our rationale is supported by the well-established notion that the root cause of drug and non-drug addictions (i.e. Reward Deficiency Syndrome [RDS]), at least in adults, involve dopaminergic dysfunction and heightened stress. This proposition involves coupling the Genetic Addiction Risk Score (GARS) with a subsequent polymorphic matched genetic customized Pro-Dopamine Regulator known as KB220ZPBM (Precision Behavioral Management). Induction of dopamine homeostasis will be clinically beneficial in attempts to combat BUD for at least three reasons: 1) During detoxification of alcoholism, the potential induction of dopamine regulation reduces the need for BZDs; 2) A major reason for BZD abuse is because people want to achieve stress reduction and subsequently, the potential induction of dopamine regulation acts as an anti-stress factor; and 3) BUD and OUD are known to reduce resting state functional connectivity, and as such, potential induction of dopamine regulation enhances resting state functional connectivity. Future randomized placebo-controlled studies will investigate this forward thinking proposed novel modality.

## Introduction

Benzodiazepines (BZDs) like Xanax, Valium and Ambien are household names. They are widely popular and widely prescribed. *Psych Central* reports that in 2013, Xanax, a benzodiazepine, was the most commonly prescribed drug [1]. Other benzodiazepines, Ativan and Valium, came in fifth and ninth in popularity [2]. The Drug Enforcement Agency reports the number of prescriptions written in 2011 for popular benzodiazepines includes: Alprazolam (Xanax): 49 million prescriptions; Lorazepam (Ativan): 27.6 million prescriptions; Clonazepam (Klonopin): 26.9 million prescriptions; Diazepam (Valium): 15 million prescriptions; and Temazepam (Restoril): 8.5 million prescriptions [3,4]. More than 30 percent of overdoses involving opioids also involve benzodiazepines. Benzodiazepines (sometimes called “benzos”) work via different mechanisms (i.e. stimulating GABA synthesis, inhibiting reuptake, mimicking GABA, etc.) to calm or sedate a person by raising the level of the inhibitory neurotransmitter GABA in the brain.

**Figure 1.**
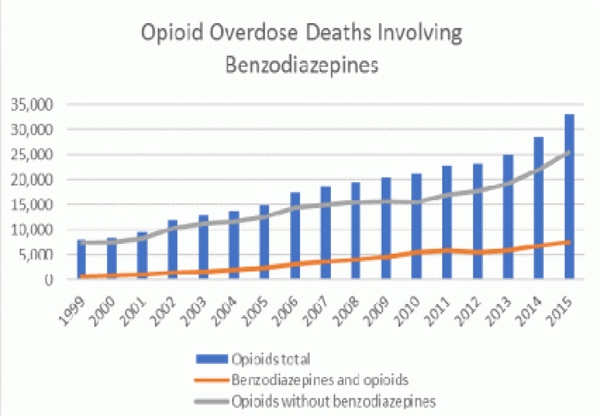
Control and prevention (CDC). Multiple cause of death, 1999–2015. (Source: Centers for Disease)

Doctors prescribe both BZDs and opioids simultaneously based on the diagnosed need to medicate their respective symptoms without a deeper understanding of either conflicting or overlapping mechanisms of action. Every day, more than 115 Americans die after overdosing on opioids [5]. However, between 1996 and 2013, the number of adults who filled a benzodiazepine prescription increased by 67%, from 8.1 million to 13.5 million [6]. Unfortunately, the quantity obtained also increased from 1.1 kg to 3.6 kg lorazepam-equivalents per 100,000 adults. Undoubtedly, combining opioids and benzodiazepines can be unsafe because both types of drug sedate users and suppress breathing-the cause of overdose fatality-as well as impair cognitive functions. In 2015, 23% of people who died of an opioid overdose also tested positive for benzodiazepines (Figure 1) [7]. In a study of over 300,000 continuously insured patients receiving opioid prescriptions between 2001 and 2013, the percentage of persons also prescribed benzodiazepines rose to 17% in 2013 from 9% in 2001 [8]. suggested that people concurrently using both drugs are at higher risk of visiting the emergency department or being admitted to a hospital for a drug-related emergency.

Previous studies also highlighted the dangers of co-prescribing opioids and benzodiazepines. A cohort study in North Carolina found that the overdose death rate among patients receiving both types of medications was 10 times higher than among those only receiving opioids [9]. Moreover, Gomes et al. [10]. reported that of the overdose deaths in people prescribed opioids for non-cancer pain in Canada, 60% also tested positive for benzodiazepines [10]. Furthermore, Park et al. [11] found that American veterans with an opioid prescription receiving a benzodiazepine prescription were at increased risk of drug overdose death in a dose-response fashion.

In 2016, the Centers for Disease Control and Prevention (CDC) issued new guidelines for the prescribing of opioids [12]. CDC recommends that clinicians avoid prescribing benzodiazepines concurrently with opioids whenever possible. Both prescription opioids and benzodiazepines now carry FDA “black box” warnings on the label highlighting the dangers of using these drugs together.

## Benzodiazepine use disorder (BUD)

As stated above, BZD abuse and dependence is a serious clinical problem in not only the methadone maintenance treatment (MMT) population but also with opioids in general. Estimates of the prevalence of BUD among MMT patients range from 21% to 66% [13–20], with roughly half of patients starting BZD use after entering MMT [21]. It is noteworthy that MMT patients with BUD are likely to be using other drugs, including heroin, engage in high risk behaviors, and have increased rates of depression, anxiety, and global psychopathology [22]. It is unfortunate but factual that women in MMT who misuse BZDs during pregnancy have babies of significantly lower birth weight than those who do not [23]. In fact, MMT patients with BUD have an 8-fold likelihood of death compared to other MMT patients, as suggested by Caplehorn and associates [24]. Unfortunately, BUD in MMT programs is widespread and is associated with a negative impact on treatment outcomes [25,26].

**Figure 2.**
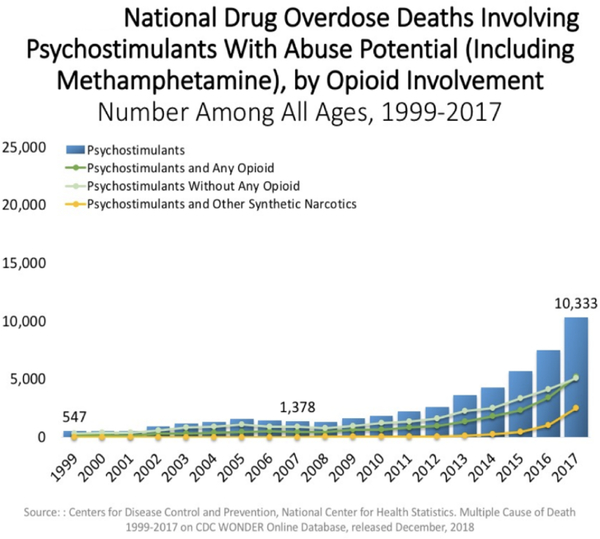
Oversdose deaths psychostimulants and opioids

**Figure 3.**
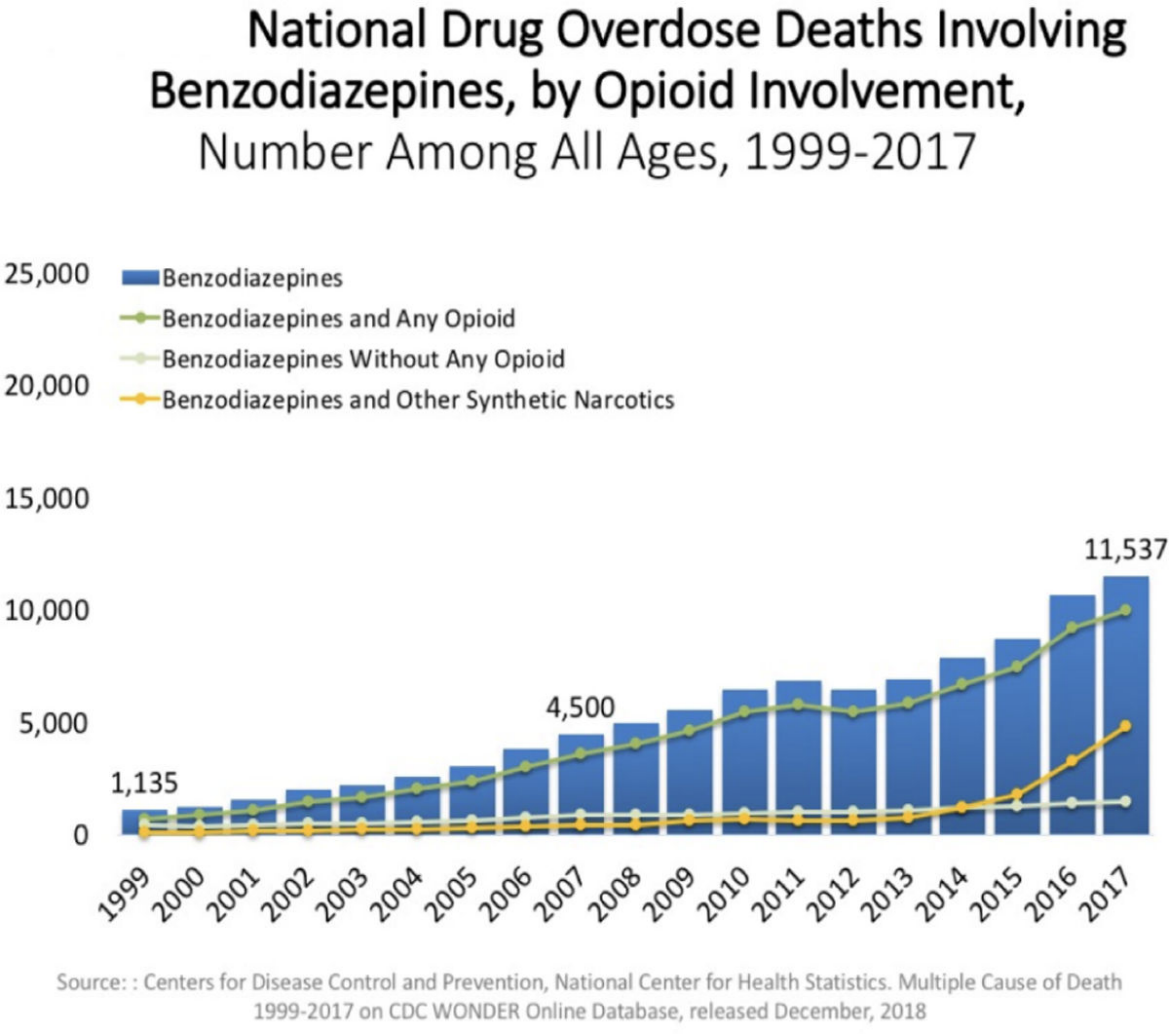
Oversdose deaths Benzodiazepines and opioids

In fact, on a national basis, opioid overdose is increased in either psychostimulant combinations (Figure 2) or even in BZD combination with opioids (Figure 3).

It is well established that Gamma-aminobutyric acid (GABA) type A receptors are a family of ligand-gated ion channels responsible for inhibitory regulation of the central nervous system, especially impacting reward circuitry and attenuation of dopamine release at the VTA-Accumbens [27]. BZDs bind directly to a specific modulatory site that is present on GABA_A_ receptors [28,29], which enhances the effect of the inhibitory neurotransmitter, but does not open the chloride channel in the absence of GABA. Moreover, chronic exposure to BZDs leads to modulation of GABAergic neurotransmission, resulting in symptoms of tolerance, dependence and withdrawal. It is believed that these effects of BZDs are linked directly to GABA_A_ receptor subtypes [30], and the withdrawal syndrome may in part be mediated by calcium channels [31] and glutamatergic [32] mechanisms.

It is well-known that discontinuation of therapeutic BZD treatment has generally supported the approach of a slow, gradual taper [33]. There is a plethora of many small clinical trials involving many pharmaceuticals including: propranolol [34]; buspirone [35]; progesterone [36]; Hydroxyzine [37]; Carbamazepine [38]; Trazodone and valproate [39]; Cyamemazine [40]; and flumazenil [41]. However, no clearly effective pharmacotherapy for the treatment of BZD use disorder has been identified. In addition, even slow outpatient detoxification has not been accepted in clinical practice, and as such, there is no known optimal strategy for BUD globally [42].

One therapeutic approach, which has been studied and is considered to be beneficial clinically to offset BZDS withdrawal and other side effects, is Gabapentin. Briefly, Gabapentin, is a structural analogue of the neurotransmitter GABA [43] and while it is not a GABA-mimetic agent [44], it has been shown to increase synaptic GABA levels [45,46], Gabapentin binds to the α_2_δ−1 and α_2_δ−2 subunits of voltage-gated calcium channels [47–49] and inhibits calcium currents [50–52], which leads to attenuation of postsynaptic excitability [53,54], potentially leading to a reduction of dopamine at the Nucleus accumbens (NAc). However, there have been no chronic studies in humans related to the effects of chronic Gabapentin and dopaminergic transmission. There are some studies which suggest that Gabapentin has anxiolytic [55,56] and sedating properties [57].

With this rationale, Mariani et al. [58]carried out an eight-week randomized double-blind placebo-controlled outpatient pilot trial using Gabapentin to help treat BUD in methadone patients. They found no significant differences on BZD use outcomes (amount BZD per day (Mann-Whitney U = 27, p = .745), abstinent days per week (U = 28, p = .811)) and CIWA-BZD scale (U = 29.0, p = .913). The authors conclude that Gabapentin was not found to differ from placebo, although the small sample recruited for this trial may have limited the ability to detect a difference.

## The need for “dopamine homeostasis”

Despite the risk of BUD on MMT patients, to date there is no clearly effective treatment strategy for managing this clinical issue. Therefore, we are proposing a novel approach we term “Precision Addiction Management” [59] and “Precision Behavioral Management.”

Every day in America, millions of people from all walks of life are unable to combat their fatal romance with getting high. For many caught in all kinds of addictive behaviors, this “high” may be just experiencing feelings of well-being [60]. Importantly, while it is widely accepted that dopamine is a major neurotransmitter implicated in behavioral and substance addictions, including BUD, there remains controversy about how to modulate dopamine clinically. One important question relates to genetic antecedents of hypodopaminergia, placing people at high risk for all addictive behaviors including BUD [61]. We believe that early genetic identification of hypodopaminergia could translate to early intervention. A prudent approach may be biphasic; a short-term blockade followed by long-term dopaminergic upregulation, achieving dopamine homeostasis [62].

The goal of treatment would be to enhance brain reward functional connectivity volume, targeting reward deficiency and the stress-like anti-reward symptomatology of addiction [63,64]. Such phenotypes can be characterized using the Genetic Addiction Risk Score (GARS) [65]. Dopamine homeostasis may thus be achieved via “Precision Addiction Management” (PAM) [58,66], also known as “Precision Behavioral Management” (PBM); the customization of neuronutrient supplementation based on the GARS test result, along with a behavioral intervention.

## Dopamine homeostasis and BUD

We are proposing herein that induction of dopamine homeostasis will be clinically beneficial in attempts to combat BUD for at least three reasons: 1) During detoxification of alcoholism, the potential induction of dopamine regulation reduces the need for BZDs; 2) A major reason for BZD abuse is that people want to achieve a stress reduction, and subsequently, potential induction of dopamine regulation acts as an anti-stress factor; and 3) BUD and OUD are known to reduce resting state functional connectivity, and as such, potential induction of dopamine regulation enhances resting state functional connectivity.

## Common mechanisms in detoxification

It is well known that a major use of BZDs is to help reduce alcohol-induced withdrawal symptoms because of inhibition of excitatory neuronal activity. Of interest, it is also known that dopamine and morphine significantly reduce alcohol-induced withdrawal symptoms, suggesting common mechanisms [67,68]. In 1988, Blum et al. [69] reported on results of a double-blind investigation of a Pro-dopamine regulator (now identified as ‘KB220’ and evolved variants) for facilitating improvement in a 30-day inpatient alcohol and drug rehabilitation center. KB220 is uniquely designed to elevate levels of enkephalin(s), serotonin, catecholamines, and GABA, which are believed to be functionally deficient in alcoholics. The KB220 patients, as compared to the control group, had a lower building up to drink score (1 vs 2); required no PRN BZDs (0% vs 94%); ceased tremoring at 72 h, as compared to 96 h; and had no severe depression on the MMPI, in contrast to 24% of control group. The take home message here is that KB220, a known Pro-dopamine regulator [70], significantly reduced the need for BZDs in residential treatment for alcoholism. These results suggest the importance of potentially achieving dopamine balance, especially during detoxification as we observed with opioid withdrawal [71].

## Dopaminergic anti-stress potential

While it is accepted that up regulation of GABA_A_ receptors results in an attenuation of stress in animal models [72] as well as humans [73], the role of dopamine as an anti-stress molecule is also known [74]. In fact, electrophysiological experiments reveal that aversive stimuli block putative VTA dopamine neuron firing [75–78], Interestingly, microdialysis investigations examining extracellular dopamine and its metabolites found a robust dopaminergic increase during stress in VTA projection targets. Specifically, stressors such as restraint, foot-shock, tail pinch/shock, and social threat, increase extracellular dopamine in the NAc and mPFC [79–82]. Moreover, studies reveal long-lasting neuroadaptive changes on VTA dopamine neurons after a single stress exposure [83]. According to Holly & Miczek [83], these studies highlight that acute stress can modify VTA dopamine neuron responsivity. Understanding the powerful role of dopamine during stressful conditions, and the known fact that hypodopaminergia (possibly genetically) is a culprit for addiction relapse [84,85], and an inability to cope with even family stress in alcoholics [86], provides the rationale for suggesting that KB220 variants could have anti-anxiety effects via dopamine regulation or induction of homeostasis, which could combat BUD. Comings et al. [86] reported that DRD2 A1 genotype–phenotype associations depend on the magnitude of stress exposure, and these results lend support to the view that variability in DRD2 study outcomes may in part be explained by this geneenvironment interaction. Specifically, Volkow’s group showed through PET scans that drug abusers have marked decreases in dopamine D2 receptors and in dopamine release. Moreover, this decrease in dopamine function or hypodopaminergia is associated with reduced regional activity in orbitofrontal cortex (involved in salience attribution; its disruption results in compulsive behaviors), cingulate gyrus (involved in reinstatement of drug abuse) and dorsolateral prefrontal cortex (involved in executive function and decision -making). With brief snapshot of the involvement of Dopamine as an anti-stress molecule, our laboratory found significant evidence regarding the pro-dopamine regulator KB220Z to act as an anti–anxiety agent [87]. Along these lines of investigation, Blum et al., in a published double-blinded placebo-controlled study, found the complex KB220 in alcoholics and polydrug abusers attending an in-patient chemical dependency program, showed significant anti-anxiety effects. Specifically, in 62 alcoholic and polydrug abusers, Blum’s group utilized skin conductance level (SCL) to evaluate stress responses. Patients administered KB220 had a significantly reduced stress response as measured by SCL, compared to patients administered placebo. A two factor ANOVA yielded significant differences as a function of Time (p< 0.01). These results support the notion that regulation of dopamine and possible induction of its homeostasis in chemical dependent patients may improve treatment response in heroin in-patient treatment setting by reducing stress related behaviors and warrants further investigation [87].

## Induction of enhanced functional connectivity

Stein’s group from NIDA has clearly established the importance of resting state functional connectivity in addiction medicine [88,89]. Assessing network dynamics via resting-state functional connectivity (rsFC) enables a better understanding of how reductions in rsFC and circuit connectivity link into dopaminergic dysfunction and reward processing and liability to addictive–like behavior. There are many examples showing that drugs of abuse including nicotine, cocaine, alcohol, heroin and even BZDs tend to reduce rsFC [90]. In fact, it was found that oxazepam decreased connectivity between, for example, the modulated amygdala and temporal cortex, whereby L-dopa also altered functional connectivity. With this background it seems prudent that one way to improve clinical outcomes is to target potential enhancement of rsFC in both naïve animals [64] and heroin–abstinent humans [63]. We have learned from the work of Febo et al. [64], that the pro-dopaminergic complex KB220Z significantly enhances, above placebo, functional connectivity between reward and cognitive brain areas in the rat. Importantly, these include the nucleus accumbens, anterior cingulate gyrus, anterior thalamic nuclei, hippocampus, prelimbic and infralimbic loci. Moreover, significant functional connectivity increased brain connectivity volume recruitment (potentially neuroplasticity), and dopaminergic functionality across the brain reward circuitry. Most importantly, increases in functional connectivity were specific to these regions and were not broadly distributed across the brain. In other work from our laboratory, along with Mark Gold and associates from China, we evaluated the role of KB220Z on neural pathways in reward circuitry of abstinent genotyped heroin addicts. In part, the rational was based on the work of Willuhn et al. [91]. who reported cocaine use and even non-substance-related addictive behavior increases as dopaminergic function is reduced? It has been shown by Zhang et al. [92] that rsFC is disruptive in abstinent heroin addicts. We hypothesized that treatment strategies, like dopamine agonist therapy with substances like KB220Z, may be a prudent approach to relapse prevention in abstinent heroin probands. We investigated the effect of KB220Z on reward circuitry of 10 heroin addicts undergoing protracted abstinence (average 16.9 months). Specifically, we found that in a randomized placebo-controlled crossover study of KB220Z, five subjects completed a triple-blinded experiment. In addition, nine subjects were genotyped utilizing the GARS test. KB220Z induced an increase in **B**lood **O**xygen **L**evel **D**ependent (‘BOLD’) activation in caudate-accumbens-dopaminergic pathways compared to placebo following 1-hour acute administration. Moreover, KB220Z induced an increase in functional connectivity in a putative network that included the dorsal anterior cingulate, medial frontal gyrus, nucleus accumbens, posterior cingulate, occipital cortical areas, and cerebellum.

## Summary

Prescriptions for Benzodiazepines (BZDs) have risen continually, and according to national statistics, its combination with opioids has also increased since 1999 up to 2019 (time of this writing). Benzodiazepines (sometimes called “benzos”) work to calm or sedate a person by raising the level of the inhibitory neurotransmitter GABA in the brain. In terms of neurochemistry, BZDs act at the GABA_A_ receptors to inhibit excitatory neurons reducing VTA glutaminergic drive to reduce dopamine release at the accumbens.

While Benzodiazepine Use Disorder (BUD) is very difficult to treat, partly because BZD are used to reduce anxiety and paradoxically induce a hypodopaminergia, we are proposing a paradigm shift. Instead of simply targeting chloride channel direct GABA_A_ receptors for replacement or substitution therapy, we propose the induction of dopamine homeostasis. Our rationale is supported by the well-established notion that the root cause of drug and non-drug addictions (i.e. Reward Deficiency Syndrome [RDS]), at least in adults, involve dopaminergic dysfunction and heightened stress; an epigenetic phenomenon.

This proposition involves coupling the Genetic Addiction Risk Score (GARS) with a subsequent polymorphic matched genetic customized Pro-Dopamine Regulator known as KB220ZPBM. Induction of dopamine homeostasis will be clinically beneficial in attempts to combat BUD for at least three reasons: 1) During detoxification of alcoholism, the potential induction of dopamine regulation reduces the need for BZDs; 2) A major reason for BZD abuse is because people want to achieve stress reduction and subsequently, potential induction of dopamine regulation acts as an anti-stress factor, and; 3) BUD and OUD are known to reduce resting state functional connectivity, and as such, potential induction of dopamine regulation enhances resting state functional connectivity. The experimental support for the neuropharmacological and neurological basis of KB220 can be gleaned from the work of Blum et al. [93]. Future studies will investigate through randomized placebo-controlled studies this forward thinking proposed novel modality.
